# A psychometric approach to assessments of problematic use of online pornography and social networking sites based on the conceptualizations of internet gaming disorder

**DOI:** 10.1186/s12888-020-02702-0

**Published:** 2020-06-19

**Authors:** Manuel Mennig, Sophia Tennie, Antonia Barke

**Affiliations:** 1 Division of Clinical Psychology and Psychotherapy, Philipps-University Marburg, Gutenbergstrasse 18, 35032 Marburg, Germany; 2 Clinical and Biological Psychology, Catholic University of Eichstätt-Ingolstadt, Eichstätt, Germany

**Keywords:** Social networking, Online pornography, Cyberpornography, Problematic internet use, Questionnaire, Validation

## Abstract

**Background:**

The problematic use of online gaming, social networking sites (SNS) and online pornography (OP) is an evolving problem. Contrary to the problematic use of SNS and OP, Internet gaming disorder (IGD) was included in the new edition of the *Diagnostic and statistical manual of mental disorders* (DSM-5) as a condition for further study. The present study adapted the criteria for IGD to the problematic use of SNS and OP by modifying a validated questionnaire for IGD (Internet Gaming Disorder Questionnaire: IGDQ) and investigating the psychometric properties of the modified versions, SNSDQ and OPDQ.

**Methods:**

Two online samples (SNS: *n* = 700, 25.6 ± 8.4 years, 76.4% female; OP: *n* = 700, 32.9 ± 12.6 years, 76.7% male) completed the SNSDQ/OPDQ, the Brief Symptom Inventory (BSI) and the short Internet Addiction Test (sIAT) and provided information on their SNS/OP use. Standard item and reliability analyses, exploratory and confirmatory factor analyses and correlations with the sIAT were calculated. Problematic and non-problematic users were compared.

**Results:**

The internal consistencies were ω_ordinal_ = 0.89 (SNS) and ω_ordinal_ = 0.88 (OP). The exploratory factor analyses extracted one factor for both questionnaires. Confirmatory factor analyses confirmed the results. The SNSDQ/OPDQ scores correlated highly with the sIAT scores and moderately with SNS/OP usage time. Of the users, 3.4% (SNS) and 7.1% (OP) lay above the cutoff for problematic use. Problematic users had higher sIAT scores, used the applications for longer and experienced more psychological distress.

**Conclusion:**

Overall, the results of the study indicate that the adaption of the IGD criteria is a promising approach for measuring problematic SNS/OP use.

## Background

In 2017, 3.5 billion people used the Internet [1]. Of the many ways of using it, online gaming, social networking sites (SNS) and online pornography (OP) are especially popular. All of these applications are under investigation, since their problematic use seems to be linked to psychological distress and problems with work, academic performance and interpersonal relationships [2–7]. With its inclusion in the appendix of the fifth edition of the *Diagnostic and statistical manual of mental disorders* (DSM-5), *Internet gaming disorder* (IGD) was recognized as a disorder warranting further investigation [8]. This was the first step towards defining standardized criteria for it. The 9 criteria are based on those for substance use disorders and gambling disorder and have to be fulfilled for the last 12 months: (1) preoccupation with gaming, (2) withdrawal when unable to game, (3) tolerance, (4) failure to stop/reduce the amount of gaming, (5) giving up other activities in favour of gaming, (6) continuing to play despite problems, (7) deceiving others about its amount, (8) gaming to escape adverse moods and (9) jeopardizing an important relationship, one’s occupation or one’s education because of gaming.

While IGD was included in the DSM-5 as a condition for further study, the problematic use of SNSs and OP was not. Petry and O’Brien (2013) [9] argue that there is a lack of empirical evidence and inconsistency in studies investigating these issues (SNS and OP). Nevertheless, there is an ongoing debate about the existence, classification and diagnosis of the problematic use of specific Internet applications like SNSs or OP [10] and a growing number of studies indicate the relevance of problematic use of SNS and OP [3, 5, 11, 12], not least due to their association with increased levels of psychological distress. This may even include symptoms of psychiatric disorders like depression, anxiety disorders, attention deficit and hyperactivity disorder or obsessive-compulsive disorder [2, 11, 13–15].

### Assessment of problematic SNS and OP use

There are a number of different diagnostic instruments to assess a problematic use of SNS and OP. Most of them are either based on the diagnostic criteria for behavioural addictions (SNS: e.g. Bergen Social Media Addiction Scale [16] | OP: e.g. Problematic Pornography Consumption Scale [17]) or the Internet Addiction Test [18] (SNS: e.g. Addictive Tendencies Towards SNSs Scale [19] | OP: sIAT-sex [20]). Note, that this is by no means an exhaustive enumeration of all diagnostic instruments. For a detailed overview see Andreassen (2015) [2] for SNS and Wéry & Billieux (2017) [21] for OP. There is no shortage of well-validated instruments, but the following problems still remain: (i) different theoretical conceptualisations of problematic SNS and OP use with the consequence (ii) that no unified, standardized criteria are available to assess problematic use of the three most important specific online applications (Gaming, SNS, OP) in a comparative manner.

The most recent theoretical model for specific Internet-use disorders is the I-PACE model [22]. It is based on empirical findings and integrates previous theoretical considerations from other models in the field of behavioral addictions, like the Syndrome Model [23] or the Components Model of Addiction [24]. The I-PACE model hypothesizes that the aetiology of problematic use is similar for different Internet applications. Therefore, it suggests the application of uniform diagnostic criteria to all applications, thereby standardizing the diagnostic criteria and allowing comparisons of their prevalence rates. Since the American Psychiatric Association already proposed standardized criteria for IGD, it suggests itself to apply these criteria to the problematic use of other internet applications and there are several researchers who agree with this approach [25–27]. Some studies have already used this approach to develop psychometric tools to assess problematic internet use [26, 28, 29] However, to the best of the authors’ knowledge, there is only one study that used this approach for the problematic use of SNS [27] and none for the problematic use of OP.

### Aim of the present study

Therefore the aim of this study was to examine to which extent the conceptualization of the Internet Gaming Disorder can be adapted to the problematic use of SNS and OP. Petry et al. (2014) [30] – who were members of the Substance Use Disorder Work group that recommend to include IGD in the DSM-5 - published a questionnaire (Internet Gaming Disorder Questionnaire: IGDQ) to assess IGD. For this study, we used the German version, which was validated by Jeromin, Barke and Rief (2016) [31] and adapted it for problematic SNS and OP use by rephrasing the items (for details see “Measures” section). In order to assess and evaluate to what degree the concept of the IGD can furnish a useful starting point for the assessment of problematic use of SNS and OP, we investigated the psychometric properties of the two modified versions, the SNSDQ and OPDQ.

## Methods

### Participants and procedure

The data were collected via an online survey (October 2017 – January 2018). The link to the questionnaire was posted to general (e.g. reddit) and application-specific Internet forums (e.g. facebook groups), SNS and mailing lists. At the outset, the participants specified whether they mainly use SNS or OP and were redirected to the corresponding questionnaire (SNS/OP). As an incentive, participants could win one of five gift vouchers for an online store (voucher value: €20). The inclusion criteria were: informed consent, age ≥ 18 years. Exclusion criteria were: no native speaker (German), percentage of online time spent using SNSs/OP ≤5%.

### SNS subsample

A total of 939 participants fulfilled the inclusion criteria. Of these, 239 (25.45%) had to be excluded: 228 because they had missing data for the SNSDQ, 7 because they failed to provide serious information (e.g. Klingon as their native language) and 4 because they had an unrealistically fast answering time (2 SDs below the mean time). In the end, data from 700 participants were analysed (Table 1).

**Table 1:** Characteristics of the SNS and OP samples

	*SNS sample (n = 700)*		*OP sample (n = 700)*	
	M	SD	M	SD
Age (years)	25.6	8.4	32.9	12.6
Internet time in a typical week (h)	20.9	14.8	21.9	15.6
Duration of a typical session (h)	2.9	5.6	2.8	4.7
SNS/OP use in a typical week (h)	9.4	10	3.9	6.1
SNSDQ/OPDQ score	1.2	1.5	1.5	1.7
sIAT score	23.6	7.3	22.3	7.9
BSI total score	9.8	16.7	25.6	27.6
	Men*	*Women*	*Men*	*Women*
Sex	156	535	537	156

* *Note.* SNS: *n* = 9 (1.3%), OP: *n* = 7 (1%) preferred not to specify their sex

### OP subsample

A total of 1858 participants met the inclusion criteria. Of these, 669 (36.01%) had to be excluded: 630 because they had missing data for the OPDQ, 25 because they provided obviously false information, 9 because of an unrealistically fast answering time and 5 due to comments suggesting that they had failed to understand the survey. To increase the statistical comparability of the two subsamples (SNS/OP), a random sample of 700 participants was drawn from the remaining 1189. Finally, data from 700 participants were analysed (Table 1).

### Measures

#### Socio-demographic information

Information regarding gender, age, education, employment and relationship status was collected.

#### Information regarding general and specific internet use

The participants reported how much time (hours) they spend online in a typical week. In addition, they provided specific information regarding their SNS or OP use, such as which SNS/OP sites they mostly use and how long they use SNSs or OP (hours/week).

#### Problematic use

The tendency of problematic SNS or OP use was assessed with the German versions of the SNSDQ and OPDQ. These questionnaires are modified versions of the IGDQ. The IGDQ consists of nine items, which reflect the corresponding DSM-5 criteria for IGD. It has a dichotomous response format consisting of ‘no’ (0) and ‘yes’ (1). The score is obtained by adding the responses (score range: 0–9). A score of ≥ 5 was defined as the cutoff for receiving a diagnosis of IGD [30]. For its adaptation regarding SNS and OP, the original items were rephrased by replacing all references to online gaming with references to SNS or OP. For example, ‘Do you feel restless, irritable, moody, angry, anxious or sad when attempting to cut down or stop using SNS or when you are unable to use SNS?’ instead of ‘Do you feel restless, irritable, moody, angry, anxious or sad when attempting to cut down or stop gaming or when you are unable to play?’

#### Short internet addiction test

The sIAT is a short version of the Internet Addiction Test and consists of 12 statements expressing possible symptoms of problematic Internet use (e.g. ‘How often do you find yourself saying “just a few more minutes” when online?’) [18]. For our study, we used the validated German version and rephrased the items for SNS and OP use (e.g. ‘How often do you try to cut down the amount of time you spend watching online pornography and fail?’) [32]. The participants have to rate the frequency with which they experienced each symptom in the last week on a 5-point scale ranging from 1 (‘never’) to 5 (‘very often’). In the resulting sum score (12–60 points), higher scores indicate more problematic use. The internal consistencies of the adapted scales in the present study were good (SNS: ω = 0.88 | OP: ω = 0.88).

#### Brief symptom inventory

The German version of the Brief Symptom Inventory (BSI) was used to identify clinically relevant symptoms of the participants [33, 34]. The BSI consists of 53 statements expressing symptoms of psychological distress (e.g. ‘In the last 7 days, how much were you distressed by feeling tense or keyed up?’). The items are answered on a 5-point scale ranging from 0 (‘not at all’) to 4 (‘extremely’). The total score ranges between 0 and 212, with higher scores indicating a higher level of distress. The internal consistency in the present samples was excellent, with ω = 0.96 (SNS) and ω = 0.96 (OP).

#### Data analysis

Statistical analyses were conducted using SPSS 24 (IBM SPSS Statistics), SPSS Amos, R version 3.5.1 [35] and FACTOR for the exploratory factor analysis (EFA) [36]. For the standard item analyses for each questionnaire, the SNSDQ and the OPDQ, item difficulties and item–total correlations were calculated. As a measure of reliability, coefficient omega or ordinal omega (in case of binominal data) were computed. These coefficients are recommended as a more accurate alternative to Cronbach’s alpha, especially when the assumption of tau-equivalence is violated [37–40]. Regarding validity, we investigated the factor structures by conducting EFAs and confirmatory factor analyses (CFA). For these, each sample (SNS and OP) was randomly divided into two subsamples (SNS1, SNS2 and OP1, OP2; each subsample: *n* = 350). The subsamples SNS1 and OP1 were used for the EFAs and SNS2 and OP2 for the CFAs. All other calculations are based on the total samples. To test whether the subsamples differed in key variables (age, SNSDQ/OPDQ score), independent t tests were performed. To ascertain the data’s suitability for EFA, the Kaiser–Meyer–Olkin test (KMO) and Bartlett’s test of sphericity were employed. Due to the dichotomous response format of the SNSDQ and the OPDQ, the EFAs followed Jeromin et al. (2016) [31] and used tetrachoric correlations as input and unweighted least squares as the estimation method [41]. The number of factors to be extracted was determined using Velicer’s MAP test [42].

A CFA was performed on SNS2 and OP2 to test the factor solution. The model parameters were estimated using maximum likelihood estimations. Due to the violation of the normality assumption Bollen-Stine Bootstrapping was applied [43]. To evaluate the model fit, the comparative fit index (CFI), root mean square error of approximation (RMSEA) and standardized root mean square residual (SRMR) were calculated. According to Hu and Bentler (1999) [44], the cutoff criteria for an acceptable model fit are a CFI of > 0.95, an RMSEA between 0.06 and 0.08 and an SRMR of < 0.08.

Bivariate relationships between the SNSDQ and OPDG scores and the time spent using the Internet in general, the time spent using the preferred application (SNS/OP) and the sIAT scores were tested with Pearson correlations.

To give a first indication of diagnostic validity, we compared problematic users with non-problematic users. Analogously to the IGDQ, users with a score of ≥ 5 points were categorized as problematic users and all other users as non-problematic [30, 31]. Independent t tests (in the case of unequal variances: Welch’s tests) were computed to compare the groups regarding age, time spent using the Internet, time spent using their preferred application and sIAT and BSI scores. Due to the unequal group sizes, Hedges’ *g* is reported as a measure of effect size [45]. An effect of *g* = 0.20 is regarded as small, *g* = 0.50 as medium and *g* = 0.80 as large [45].

## Results

### SNS, OP and Internet use

#### SNS

**Fig. 1:**
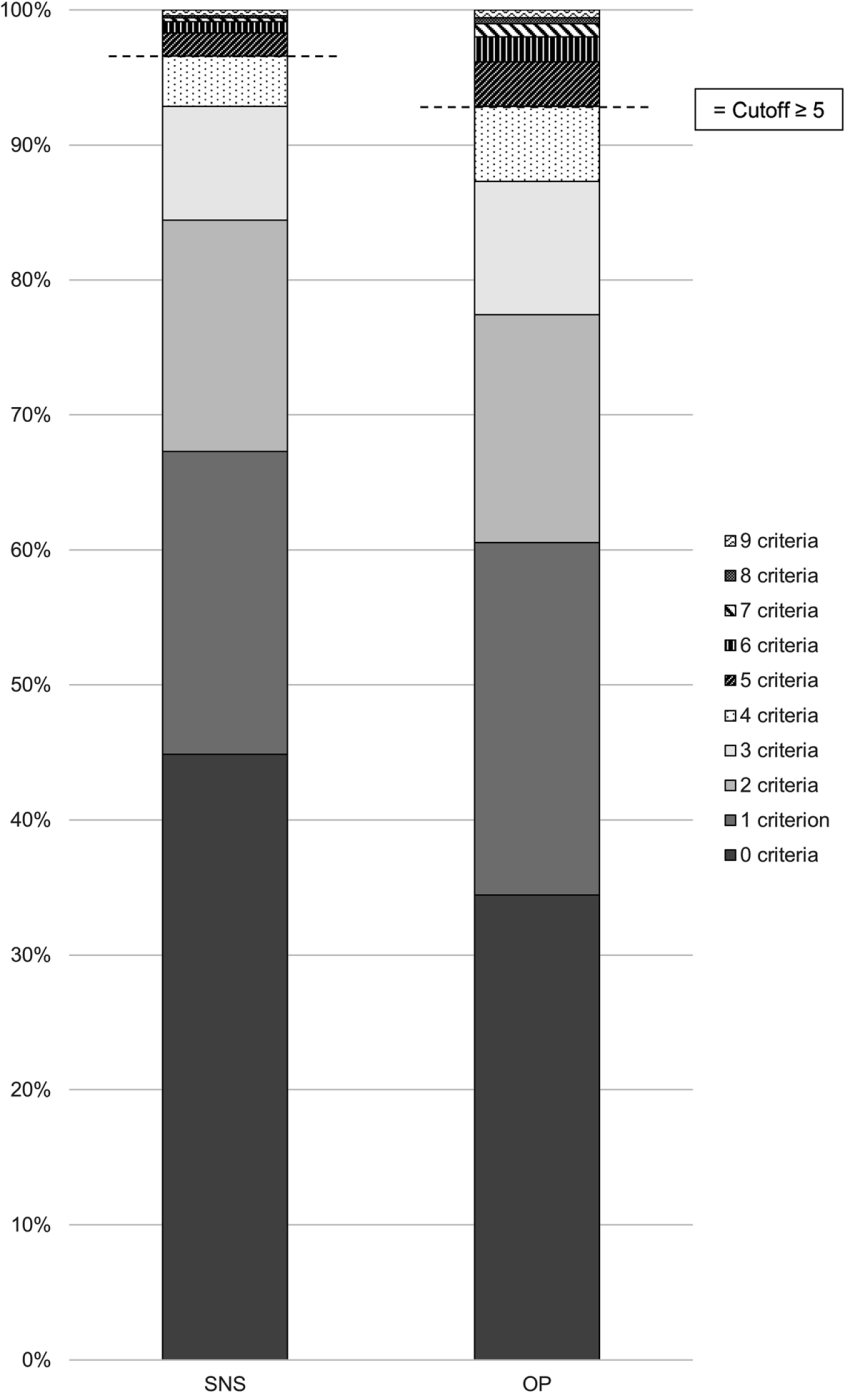
Percentage of participants fulfilling different numbers of criteria of the modified IGDQ (SNS and OP)

The participants used the Internet on average for 20.9 ± 14.8 h/week and SNSs for 9.4 ± 10 h/week (44% of the total online time), with Facebook being the most popular SNS (*n* = 355; 50.7%), followed by Instagram (*n* = 196; 28%) and YouTube (*n* = 74; 10.6%). The mean SNSDQ and sIAT scores were 1.2 ± 1.5 and 23.6 ± 7.3 points. Overall, 24 participants (3.4%) had an SNSDQ score of ≥5 points and thus lay above the cut-off for problematic use (see Fig. 1 for details). The mean BSI total score across all the participants was 9.8 ± 16.7.

#### OP

The participants used the Internet on average for 21.9 ± 15.6 h/week and consumed OP for 3.9 ± 6.1 h/week (18.9% of the total online time). The most popular form of OP was videos (*n* = 351; 50.1%), followed by pictures (*n* = 275; 39.3%) and webcams (*n* = 71; 10.1%). The mean OPDG and sIAT scores were 1.5 ± 1.7 and 22.3 ± 7.9. A total of 50 participants (7.1%) achieved an OPDQ score above the cutoff of ≥ 5 points (see Fig. 1 for details). The mean BSI score across all the participants was 25.6 ± 27.6.

#### Item analysis and internal consistency

The results of the item analyses are presented in Tables 2 and 3.

**Table 2:** Results of the item analysis and exploratory factor analysis (SNS)

*Item*	*Endorsement*	*p*_*i*_	*Factor loading*^*a*^	*r*_*itc*_	*Ordinal* ω *without item*
1. Do you spend a lot of time thinking about SNS even when you are not using them or planning when you can use them next?	29	0.04	0.71	0.34	0.88
2. Do you feel restless, irritable, moody, angry, anxious or sad when attempting to cut down or stop using SNS or when you are unable to use SNS?	38	0.05	0.74	0.38	0.88
3. Do you feel the need to use SNS for increasing amounts of time to get the same amount of excitement you used to get?	50	0.07	0.54	0.28	0.89
4. Do you feel that you should spend less time on SNS but are unable to cut back on the amount of time you spend using SNS?	193	0.28	0.70	0.39	0.89
5. Do you lose interest in or reduce participation in other recreational activities (hobbies, meetings with friends) due to using SNS?	41	0.06	0.64	0.39	0.88
6. Do you continue to use SNS even though you are aware of negative consequences, such as not getting enough sleep, being late to school/work, spending too much money, having arguments with others or neglecting important duties?	247	0.35	0.69	0.39	0.89
7. Do you lie to family, friends or others about how much you use SNS or try to keep your family or friends from knowing how much you use SNS?	21	0.03	0.71	0.34	0.88
8. Do you use SNS to escape from or forget about personal problems or to relieve uncomfortable feelings, such as guilt, anxiety, helplessness or depression?	172	0.25	0.58	0.38	0.89
9. Do you risk or lose significant relationships, or job, educational or career opportunities, because of using SNS?	32	0.05	0.78	0.38	0.88

*Note.* n = 700; ω_ordinal_ = 0.89; ^*a*^ = only EFA subsample (SNS1, *n* = 350)

**Table 3:** Results of the item analysis and exploratory factor analysis (OP)

*Item*	*Endorsement*	*p*_*i*_	*Factor loading*^*a*^	*r*_*itc*_	*Ordinal* ω *without item*
1. Do you spend a lot of time thinking about OP even when you are not online or planning when you can watch it next?	74	0.11	0.58	0.34	0.88
2. Do you feel restless, irritable, moody, angry, anxious or sad when attempting to cut down or stop watching OP or when you are unable to watch OP?	48	0.07	0.65	0.38	0.86
3. Do you feel the need to watch OP for increasing amounts of time or watch more exciting OP to get the same amount of excitement you used to get?	161	0.23	0.52	0.34	0.88
4. Do you feel that you should spend less time watching OP but are unable to cut back on the amount of time you spend watching OP?	150	0.21	0.78	0.46	0.86
5. Do you lose interest in or reduce participation in other recreational activities (hobbies, meetings with friends) due to watching OP?	50	0.07	0.81	0.47	0.86
6. Do you continue to watch OP even though you are aware of negative consequences, such as not getting enough sleep, being late to school/work, spending too much money, having arguments with others or neglecting important duties?	152	0.22	0.76	0.42	0.87
7. Do you lie to family, friends or others about how much you watch OP or try to keep your family or friends from knowing how much you watch OP?	286	0.41	0.52	0.29	0.88
8. Do you watch OP to escape from or forget about personal problems or to relieve uncomfortable feelings such as guilt, anxiety, helplessness or depression?	139	0.20	0.56	0.32	0.88
9. Do you risk or lose significant relationships or job, educational or career opportunities because of watching OP?	24	0.03	0.93	0.42	0.85

*Note. n* = 700; ω_ordinal_ = 0.88; ^*a*^ = only EFA subsample (OP1, *n* = 350)

#### SNS

For the SNS version, item 7 had the lowest endorsement (number of affirmative answers (naa) = 21), while item 6 had the highest (naa = 247). This translates into an item difficulty of *p*_i_ = 0.03 (item 7) and *p*_i_ = 0.35 (item 6), with a mean difficulty across all items of *p*_i_ = 0.13. The corrected item–total correlations ranged from *r*_itc_ = 0.28 (item 3) to *r*_itc_ = 0.39 (items 4, 5 and 6), with a mean of *r*_*i*tc_ = 0.36. The internal consistency was ω_ordinal_ = 0.89, and the scale would not have benefited from removing any item.

#### OP

In the OP version of the questionnaire, item 9 (naa = 24) had the lowest endorsement rate, whereas item 7 had the highest (naa = 286). The mean item difficulty was *p*_i_ = .17, with item 9 being the most (*p*_i_ = 0.03) and item 7 (*p*_i_ = 0.41) the least difficult. The corrected item–total correlations ranged between *r*_itc_ = 0.29 (item 7) and *r*_itc_ = 0.47 (item 5), with a mean corrected item–total correlation of *r*_itc_ = 0.38. The internal consistency was ω_ordinal_ = 0.88. Removing items would not have increased the internal consistency.

### Factor structure

The subsamples (SNS1 vs. SNS2; OP1 vs. OP2) did not differ with regard to age, gender, Internet use, SNS/OP use, sIAT, SNSDQ/OPDQ and BSI scores (see Appendix).

#### SNS

**Fig. 2:**
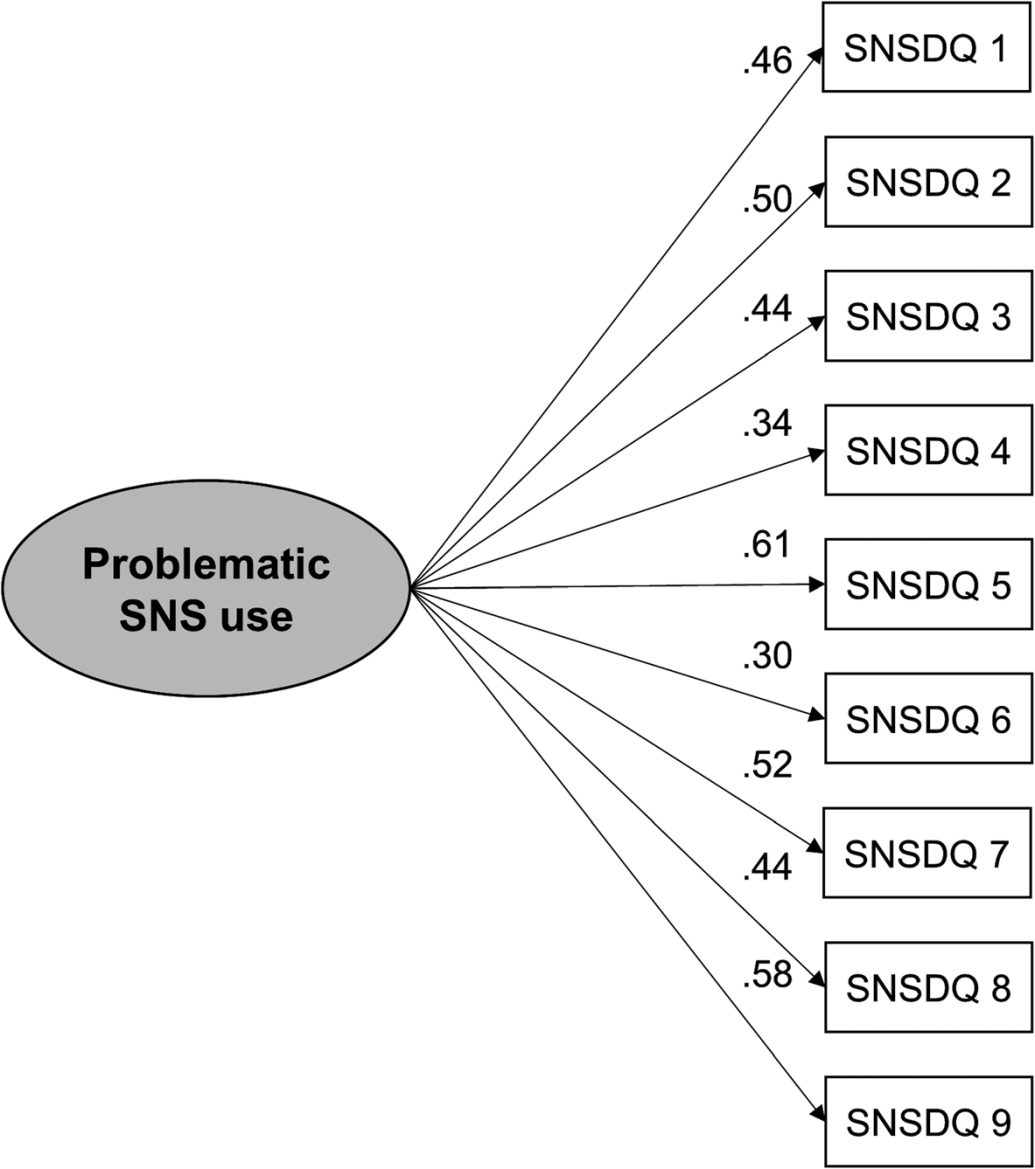
Path diagram for the confirmatory factor analysis with subsample SNS2 (*n* = 350). All path coefficients are standardized and statistically significant (*p* < 0.001)

Bartlett’s test of sphericity (Χ^2^ = 407.4, df = 36, *p* < 0.001) as well as the KMO criterion (0.74) indicated that the data were suitable for EFA. Velicer’s MAP test recommended the extraction of a single factor. This factor explained 52.74% of the total variance. The factor loadings ranged between 0.54 (item 3) and 0.78 (item 9) (Table 2). A CFA with the subsample SNS2 was calculated to test the one-factor solution. The fit indices were CFI = 0.81, RMSEA = 0.092 [CI = 0.075–0.111] and SRMR = 0.064 (for the path diagram, see Fig. 2).

#### OP

**Fig. 3:**
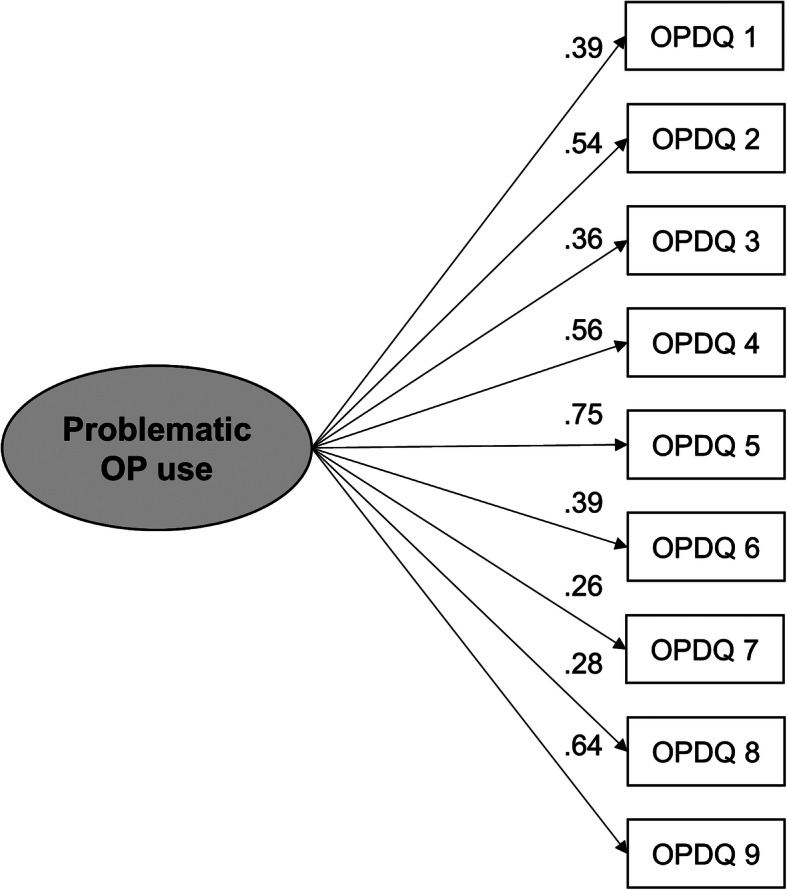
Path diagram for the confirmatory factor analysis with subsample OP2 (*n* = 350). All path coefficients are standardized and statistically significant (*p* < 0.001)

Bartlett’s test of sphericity (Χ^2^ = 455.7, df = 36, *p* < 0.001) and the KMO criterion (0.80) indicated that the data were suitable for EFA, and the MAP test suggested a one-factor solution. The extracted factor explained 53.30% of the total variance. Items 3 and 7 had the lowest factor loadings (0.52), while item 9 had the highest (0.93) (Table 3). The one-factor solution was tested with a CFA (subsample: OP2). The model fit indices were CFI = 0.87, RMSEA = 0.080 [CI = 0.062–0.099] and SRMR = 0.057 (for the path diagram, see Fig. 3).

### Correlations with SNS/OP/internet use and sIAT scores

#### SNS

The SNSDQ scores correlated with the SNS usage time (*r* = 0.32, *p <* 0.01), the weekly Internet use time (*r* = 0.16, *p <* 0.01) and the sIAT scores (*r* = 0.73, *p <* 0.01).

#### OP

The OPDQ scores correlated with the OP usage time (*r* = 0.22, *p* < 0.01) and very weakly with the Internet usage time per week (*r* = 0.08, *p* < 0.05). The highest correlation was found with the sIAT scores (*r* = 0.72, *p* < 0.01).

### Comparison of persons with problematic and non-problematic SNS/OP use

#### SNS

Compared with unproblematic users, the problematic SNS users used SNS much more and had higher sIAT scores. They also seemed to experience more psychopathological distress, but, despite the effect size of the difference, this was merely a tendency (*p* = 0.13). For details see Table 4.

**Table 4:** Comparison of the participants with problematic and non-problematic use of SNS/OP

**SNS**	*SNSDQ≥5 (n=24)*		*SNSDQ<5 (n=676)*				
	M	SD	M	SD	*t*	*df*	*g*
Age	23.25	5.39	25.73	8.47	1.423	698	.29
Internet use (h/week)	25.95	15.71	20.78	14.82	-1.678	696	.35
SNS use (h/week)	15.39	10.62	9.13	9.95	-3.017**	696	.63
sIAT score	38.79	5.68	23.02	6.77	-11.265**	698	2.34
BSI total score	16.71	22.12	9.54	16.46	-1.571	23.913^a^	.43
**OP**	*OPDQ≥5 (n=50)*		*OPDQ<5 (n=650)*				
	M	SD	M	SD	*t*	*df*	*g*
Age	33.92	13.74	32.80	12.49	-.608	698	.09
Internet use (h/week)	26.86	18.06	21.53	15.34	-2.332*	697	.34
OP use (h/week)	8.42	11.40	3.64	5.33	-2.941**	50.667^a^	.80
sIAT score	36.40	9.04	21.22	6.63	-11.618**	53.127^a^	2.22
BSI total score	57.44	40.11	23.18	24.75	-5.952**	51.909^a^	1.31

* = *p* < .05, ** = *p* < .01; ^a^Welch’s correction

#### OP

Compared with unproblematic users, participants identified as problematic OP users spent more time on the Internet in general and more time using OP, had much higher sIAT scores and experienced more psychopathological distress (Table 4).

## Discussion

In the present study, we adapted the German version of the IGDQ to the use of SNSs and OP and evaluated the psychometric properties of the modified versions in order to investigate to what extent the IGD criteria are suitable for assessing problematic use of SNS and OP.

### Item analysis

The average endorsement of the items was low for both questionnaires, which is expected and desirable given that the checklists assess criteria of problematic use in a non-clinical sample. For SNS, the most endorsed item, item 6, concerns procrastination. This appears plausible, since SNS are often used to procrastinate [46, 47]. Item 7 (deceive/cover up) received the lowest endorsement, which also seems reasonable given that many people use SNS on a daily basis and in a socially accepted manner, making lying about it unnecessary [12]. For OP, item 7 (deceive/cover up) had the highest endorsement. This is possibly the case because the social acceptance of OP is rather low even if it is used casually and many people may feel embarrassed about it [48]. The lowest endorsement was for item 9, which seems reasonable, since it implies severe consequences (risk/loss of relationships/opportunities). The corrected item–total correlations were medium for both questionnaires and above the threshold of *r*_*itc*_ = 0.30 [43]. The only exceptions were item 3 for SNS and item 7 for OP. Item 3 refers to tolerance, a criterion that is typical of substance abuse but seems to be harder to apply in the context of SNSs [49]. The low corrected item–total correlation for item 7 (OP) seems reasonable, since, as discussed, the use of OP may generally be associated with embarrassment, so deceiving others about one’s use does not discriminate well between problematic and unproblematic users.

### Reliability

The SNSDQ and the OPDG showed good internal consistencies (SNS: ω_ordinal_ = 0.89; OP: ω_ordinal_ = 0.88). The results are comparable to other questionnaires measuring problematic SNS (e.g. Bergen Social Media Scale: α = 0.88) or OP use (e.g. sIAT-sex: α = 0.88) [16, 20].

### Validity

In the course of the EFAs, a single factor was extracted for the SNS as well as the OP version of the questionnaire. This is in line with the result for the original IGDQ [31]. Item 3 had the lowest factor loading in both versions, probably because the tolerance criterion does not fit very well with the context of SNS and OP. Ultimately, the tolerance criterion originated with substance-based addictions. In that context, its meaning was much more clearly defined than with regard to the problematic use of OP, SNS or, indeed, online gaming, for which its usefulness is also discussed controversially (*pro*: [30, 50] | *contra*: [51, 52]). In the OP version, item 7 (deceive/cover up) also had a lower factor loading than the other items. This reflects the above argument regarding why the item is not so useful for differentiating between problematic and non-problematic users (37.4% of the non-problematic and 86% of the problematic users endorsed it). This indicates that the covering-up behaviour is not explicitly associated with problematic over-use measured by the OPDG but probably with social attitudes towards OP in general.

Overall, the results for the CFAs suggested that the one-factor solutions for both questionnaires are questionable and do not represent a good fit. While the SRMR was good for both models, the CFI and RMSEA were below and respectively above the cutoffs. As in the EFA, Item 6 for SNS and Item 7 for OP had particularly low factor loadings. This implies that their correlation with the respective overall scale is low and, accordingly, that their correlation with problematic usage behaviour is low. While this does not necessarily pose a problem, it is important that subsequent studies check whether these items should be revised, weighted differently or even removed.

Both questionnaires correlated strongly with the corresponding sIAT versions, indicating good convergent validity. The SNS version showed small to medium correlations with the general Internet usage and SNS usage time (per week). The OP version also showed a small correlation with the OP usage time (per week). The size of the correlations of problematic use with time spent using the respective application is in the range of those consistently reported [53–55].

To evaluate the diagnostic validity of the SNSDQ and OPDQ, we first compared observed prevalence rates with those found in other studies. For SNSs, 3.4% of the participants exceeded the cutoff, and, with regard to OP, 7.1% met the criteria for problematic use. Although comparing prevalence rates is difficult due to the multitude of different diagnostic instruments, the rates found here are comparable to some in the existing literature. In their study of a national representative sample of Hungarian adolescents, Bányai et al. (2017) [3] found a prevalence rate of 4.5% for problematic SNS use. Regarding the problematic use of OP, Giordano and Cashwell (2017) [55] reported a prevalence rate of 10.3% in a sample of American college students and Ross and colleagues (2012) [15] found a rate of 7.6% in a sample of Swedish adults.

It is important to note that no diagnosis can be made using these instruments. Firstly, neither the DSM-5 nor the ICD-11 contain any diagnoses for the problematic use of OP or SNS. Secondly, even if they did, a clinical interview by an expert would be necessary to verify the presence of clinically significant distress and functional impairment and the absence of any the exclusion criteria for the individual case, which are a requirement for a psychiatric diagnosis. Such an independent clinical judgement was not collected in the present study, so we cannot determine whether persons above the cutoff would warrant any diagnosis. However, we would consider them as possible candidates for such a diagnosis. To further investigate the diagnostic validity, we compared the users above and below the cutoff and found marked differences. Problematic users spent more time online per week (only for OP) and used their preferred application for longer. Although an increased usage time is not a sufficient criterion to infer a problematic use, several studies have found an - albeit weak - correlation between usage time and problematic use [53–55]. In addition, problematic users had much higher sIAT scores and seemed to experience a higher level of psychological distress (only for OP). Overall, these results – particularly the very large difference between the BSI total scores in the case of the problematic OP users – may be regarded as first indicators of the criterion validity of the instruments and suggest that the IGD criteria might be suitable to identify individuals with a problematic use of SNS or OP [56].

### Limitations

The study has to be considered in the light of its limitations. One limitation is that only adult participants were tested, although SNS particularly are also frequently used by adolescents [3]. A further limitation is that not all participants answered all questionnaires regarding problematic use (SNS, OP and IGD). This would have allowed a more detailed investigation of the overlapping between the problematic use of the respective applications. Moreover, only self-reported data were collected, which are prone to bias effects, like social desirability or common method variance. In addition, they did not include a clinical judgement. Considering that the aim of the self-report checklists is to identify problematic users, further studies should investigate their validity with samples of persons who are judged by clinicians to show problematic use in a clinically relevant sense. Furthermore, it is important to note that neither the criteria for a diagnosis, nor the number of items or any cut-off have been agreed. We do not intend to propose any arguments as to whether these behavioural patterns would warrant the status of a “disorder”. We rather aim to promote research into the identification of the problematic use of SNS and OP by providing a common instrument that may help with a comparative assessment and suggest using this instrument as a common starting point for such investigations, amending them as further research suggests this.

## Conclusion

As some psychometric parameters of the tested questionnaires are not satisfactory, it seems that the IGD criteria cannot simply be transferred to the problematic use of SNS/OP. Nevertheless, our overall results indicate that this is a promising starting point and support the viability of using adapted IGD criteria as a framework to assess problematic SNS/OP use. This study contributes to the research regarding measuring aspects of problematic SNS and OP use and might be a first step towards a standardized assessment and contribute to investigations of these emerging constructs. Future research should further investigate the usefulness of the DSM-5 criteria for IGD in the context of SNS/OP use.

## Data Availability

The datasets used and/or analysed during the current study are available from the corresponding author on reasonable request.

## Appendix

**Table:** **Table 5** Comparison of the subsamples with regard to age, gender, Internet use, SNS/OP use, sIAT, SNSDQ/OPDQ and BSI scores

	*Subsample 1* *(n = 350)*		*Subsample 2* *(n = 350)*				
	M	SD	M	SD	*t*	*df*	*p*
**SNS**							
Age	25.74	8.44	25.55	8.35	-0.302	698	0.76
Internet use (h/week)	20.78	14.68	21.31	15.06	0.308	696	0.75
SNS use (h/week)	8.98	8.74	9.72	11.17	0.973	657.877^a^	0.33
SNSDQ score	1.21	1.52	1.15	1.43	-0.535	698	0.59
sIAT score	23.85	7.28	23.27	7.36	-1.063	698	0.28
BSI total score	9.65	16.72	9.93	16.73	0.221	698	0.83
	*Subsample 1* *(n = 350)*		*Subsample 2* *(n = 350)*				
	M	SD	M	SD	*t*	*df*	*p*
**OP**							
Age	32.55	12.45	33.21	12.71	0.697	698	0.48
Internet use (h/week)	21.52	15.77	22.30	15.42	0.662	697	0.50
OP use (h/week)	4.00	5.81	3.96	6.36	-0.082	697	0.93
sIAT score	22.53	7.81	22.09	7.91	-0.735	698	0.46
BSI total score	26.74	29.09	24.52	25.93	-1.066	698	0.28

^a^Welch‘s correction

**Table:** **Table 6** Comparison of the gender distribution in the subsamples

	SNS		OP	
	*Subsample 1*	*Subsample 2*	*Subsample 1*	*Subsample 2*
Men	82	74	266	271
Women	262	273	80	76
Other	6	3	4	3

**SNS:**

*χ*^*2*^ = 1.63

*p* = 0.44

**OP:**

*χ*^*2*^ = 2.92

*p* = 0.86

## Abbreviations

BSI: Brief Symptom Inventory
CFA: Confirmatory Factor Analysis
CFI: Comparative Fit Index
CI: Confidence Interval
DSM-5: Diagnostic and statistical manual of mental disorders
EFA: Exploratory Factor Analysis
IGD: Internet gaming disorder (IGD)
KMO: Kaiser–Meyer–Olkin
NAA: Number of affirmative answers
OP: Online Pornography
OPDQ: Online Pornography Disorder Questionnaire
RMSEA: Root mean square error of approximation
sIAT: Short Internet Addiction Test
SNS: Social Networking Sites
SNSDQ: Social Networking Sites Disorder Questionnaire
SRMR: Standardized root mean square residual

## References

[1] ITU (2018). Number of internet users worldwide from 2005 to 2017.

[2] Andreassen CS (2015). Online social network site addiction: a comprehensive review. Curr Addict Rep.

[3] Bányai F, Zsila Á, Király O, Maraz A, Elekes Z, Griffiths MD (2017). Problematic social media use: results from a large-scale nationally representative adolescent sample. PLoS One.

[4] Griffiths MD (2011). Internet sex addiction: a review of empirical research. Addict Res Theory.

[5] Grubbs JB, Volk F, Exline JJ, Pargament KI (2015). Internet pornography use: perceived addiction, psychological distress, and the validation of a brief measure. J Sex Marital Ther.

[6] Kuss DJ, Griffiths MD (2012). Internet gaming addiction: a systematic review of empirical research. Int J Ment Health Addiction.

[7] Pontes HM, Griffiths MD (2016). Portuguese validation of the internet gaming disorder scale-short-form. Cyberpsychol Behav Soc Netw.

[8] Association AP (2013). Diagnostic and statistical manual of mental disorders: American Psychiatric Association.

[9] Petry NM, O'Brien CP (2013). Internet gaming disorder and the DSM-5. Addiction..

[10] Billieux J, Schimmenti A, Khazaal Y, Maurage P, Heeren A (2015). Are we overpathologizing everyday life? A tenable blueprint for behavioral addiction research. J Behav Addict.

[11] Alarcón R de, La Iglesia JI de, Casado NM, Montejo AL. Online porn addiction: what we know and what we don’t-a systematic review. J Clin Med 2019. doi:10.3390/jcm8010091. 10.3390/jcm8010091 PMC6352245 30650522

[12] Kuss DJ, Griffiths MD. Social networking sites and addiction: ten lessons learned. Int J Environ Res Public Health. 2017. 10.3390/ijerph14030311. 10.3390/ijerph14030311 PMC5369147 28304359

[13] Andreassen SC, Billieux J, Griffiths MD, Kuss DJ, Demetrovics Z, Mazzoni E, Pallesen S (2016). The relationship between addictive use of social media and video games and symptoms of psychiatric disorders: a large-scale cross-sectional study. Psychol Addict Behav.

[14] Hussain Z, Griffiths MD (2018). Problematic social networking site use and comorbid psychiatric disorders: a systematic review of recent large-scale studies. Front Psychiatry.

[15] Ross MW, Månsson S-A, Daneback K (2012). Prevalence, severity, and correlates of problematic sexual internet use in Swedish men and women. Arch Sex Behav.

[16] Andreassen CS, Pallesen S, Griffiths MD (2017). The relationship between addictive use of social media, narcissism, and self-esteem: findings from a large national survey. Addict Behav.

[17] Bőthe B, Tóth-Király I, Zsila Á, Griffiths MD, Demetrovics Z, Orosz G (2018). The development of the problematic pornography consumption scale (PPCS). J Sex Res..

[18] Young KS (1998). Internet addiction: the emergence of a new clinical disorder. Cyber Psychology Behavior.

[19] Wu AMS, Cheung VI, Ku L, Hung EPW (2013). Psychological risk factors of addiction to social networking sites among Chinese smartphone users. J Behav Addict.

[20] Wéry A, Burnay J, Karila L, Billieux J (2016). The short French internet addiction test adapted to online sexual activities: validation and links with online sexual preferences and addiction symptoms. J Sex Res.

[21] Wéry A, Billieux J (2017). Problematic cybersex: conceptualization, assessment, and treatment. Addict Behav.

[22] Brand M, Young KS, Laier C, Wölfling K, Potenza MN (2016). Integrating psychological and neurobiological considerations regarding the development and maintenance of specific internet-use disorders: an interaction of person-affect-cognition-execution (I-PACE) model. Neurosci Biobehav Rev.

[23] Shaffer HJ, LaPlante DA, LaBrie RA, Kidman RC, Donato AN, Stanton MV (2004). Toward a syndrome model of addiction: multiple expressions, common etiology. Harv Rev Psychiatry.

[24] Griffiths M (2009). A ‘components’ model of addiction within a biopsychosocial framework. J Subst Abus.

[25] Rumpf H-J, Bischof G, Bischof A, Besser B, Meyer C, John U (2015). Applying DSM-5 criteria for internet gaming disorder for the broader concept of internet addiction. J Behav Addict.

[26] Pontes HM, Griffiths MD (2017). The development and psychometric evaluation of the internet disorder scale (IDS-15). Addict Behav.

[27] van den Eijnden RJJM, Lemmens JS, Valkenburg PM (2016). The social media disorder scale. Comput Hum Behav.

[28] Cho H, Kwon M, Choi J-H, Lee S-K, Choi JS, Choi S-W, Kim D-J (2014). Development of the internet addiction scale based on the internet gaming disorder criteria suggested in DSM-5. Addict Behav.

[29] Pontes HM, Griffiths MD (2017). The development and psychometric properties of the internet disorder scale–short form (IDS9-SF). addicta.

[30] Petry NM, Rehbein F, Gentile DA, Lemmens JS, Rumpf H-J, Mößle T (2014). An international consensus for assessing internet gaming disorder using the new DSM-5 approach. Addiction..

[31] Jeromin F, Rief W, Barke A (2016). Validation of the internet gaming disorder questionnaire in a sample of adult German-speaking internet gamers. Cyberpsychol Behav Soc Netw.

[32] Pawlikowski M, Altstötter-Gleich C, Brand M (2013). Validation and psychometric properties of a short version of Young’s internet addiction test. Comput Hum Behav.

[33] Derogatis LR (1992). The brief symptom inventory (BSI): administration, scoring & procedures manual-II: clinical psychometric research.

[34] Franke G (2000). BSI. Brief symptom inventory - deutsche version. Manual.

[35] Core Team R. R: a language and environment for statistical computing. In: R Foundation for statistical computing. Vienna, Austria; 2018. Available online at https://www.R-project.org/.

[36] Lorenzo-Seva U, Ferrando PJ. FACTOR 9.2. Applied Psychological Measurement. 2013;37:497–8. doi:10.1177/0146621613487794.

[37] Gadermann AM, Guhn M, Zumbo B (2012). Estimating ordinal reliability for Likert-type and ordinal item response data: a conceptual, empirical, and practical guide. Pract Assess Res Eval.

[38] Peters G-J (2014). The alpha and the omega of scale reliability and validity: why and how to abandon Cronbach’s alpha and the route towards more comprehensive assessment of scale quality: Open Science framework.

[39] Trizano-Hermosilla I, Alvarado JM (2016). Best alternatives to Cronbach's alpha reliability in realistic conditions: congeneric and asymmetrical measurements. Front Psychol.

[40] Zumbo BD, Gadermann AM, Zeisser C (2007). Ordinal Versions of Coefficients Alpha and Theta for Likert Rating Scales. J Mod App Stat Meth.

[41] Holgado-Tello FP, Chacón-Moscoso S, Barbero-García I, Vila-Abad E (2010). Polychoric versus Pearson correlations in exploratory and confirmatory factor analysis of ordinal variables. Qual Quant.

[42] Velicer WF (1976). Determining the number of components from the matrix of partial correlations. Psychometrika..

[43] Bühner M (2006). Einführung in die Test- und Fragebogenkonstruktion.

[44] Hu L-t, Bentler PM (1999). Cutoff criteria for fit indexes in covariance structure analysis: conventional criteria versus new alternatives. Struct Equ Model Multidiscip J.

[45] Ellis PD (2010). The essential guide to effect sizes: statistical power, meta-analysis, and the interpretation of research results: Cambridge University press.

[46] Meier A, Reinecke L, Meltzer CE (2016). “Facebocrastination”?: predictors of using Facebook for procrastination and its effects on students’ well-being. Comput Hum Behav.

[47] Ryan T, Chester A, Reece J, Xenos S (2014). The uses and abuses of Facebook: a review of Facebook addiction. J Behav Addict.

[48] Twohig MP, Crosby JM, Cox JM (2009). Viewing internet pornography: for whom is it problematic, how, and why?. Sex Addict Compuls.

[49] Kardefelt-Winther D, Heeren A, Schimmenti A, van Rooij A, Maurage P, Carras M (2017). How can we conceptualize behavioural addiction without pathologizing common behaviours?. Addiction..

[50] Rehbein F, Kliem S, Baier D, Mößle T, Petry NM (2015). Prevalence of internet gaming disorder in German adolescents: diagnostic contribution of the nine DSM-5 criteria in a state-wide representative sample. Addiction..

[51] Kardefelt-Winther D (2014). A conceptual and methodological critique of internet addiction research: towards a model of compensatory internet use. Comput Hum Behav.

[52] Starcevic V (2016). Tolerance and withdrawal symptoms may not be helpful to enhance understanding of behavioural addictions. Addiction..

[53] Holmgren HG, Coyne SM (2017). Can’t stop scrolling!: pathological use of social networking sites in emerging adulthood. Addict Res Theory.

[54] Yu S, Wu AMS, Pesigan IJA (2016). Cognitive and psychosocial health risk factors of social networking addiction. Int J Ment Health Addiction..

[55] Giordano AL, Cashwell CS (2017). Cybersex addiction among college students: a prevalence study. Sex Addict Compuls.

[56] Sawilowsky SS (2009). New Effect Size Rules of Thumb. J Mod App Stat Meth.

